# Frequency and variability of late gadolinium "mid-wall" enhancement(MLE) depending on observer experience, image quality and underlying disease

**DOI:** 10.1186/1532-429X-13-S1-P286

**Published:** 2011-02-02

**Authors:** Christian Lücke, Daniel Karthe, Grothoff Matthias, Janine Hoffmann, Lukas Lehmkuhl, Claudia Andres, Holger Thiele, Matthias Gutberlet

**Affiliations:** 1 Radiology, Heart Center Leipzig, Leipzig, Germany

## Objective

To evaluate the ability of an inexperienced observer (IO) to reliably assess mid-wall late enhancement (MLE) and to assess the prevalence of MLE in patients with various cardiac diseases.

## Background

Late gadolinium enhancement (LGE) in cardiac MRI (cMRI) has been described as a valid tool to discriminate between cardiac diseases. It has been postulated that MLE especially occurs in patients with dilated cardiomyopathy (DCM) and myocarditis with a prognostic impact in these patients. Nevertheless, it can be difficult to differentiate true MLE from common artifacts as motion blur, partial volume effects (PVE) or wrong inversion times (TI), especially for the IO.

## Methods

We examined 97 consecutive patients (64 male, 33 female, mean age 51 ±20 years), which were referred to our department for a cMRI for various clinical indications (37 ischemic heart diseases (ICM), 16 myocarditis, 5 DCMs, 2 restrictive cardiomyopathies (RCM), 5 hypertrophic obstructive or non-obstructive cardiomyopathies, 8 congenital heart diseases (CHD), 12 patients with arrhythmias and 12 others. Besides Cine-sequences, standard LGE-sequences (IR-GRE) and phase sensitive inversion recovery (PSIR) sequences were applied and evaluated by two independent blinded observers (1 inexperienced observer (IO) with 2 months of cMRI experience and 1 experienced observer (EO) (3 years of experience). The results of the EO (Table 1) were considered as being the standard of reference.

**Table 1:** Fourfold Table of the detected Mid-wall Enhancement (MLE) by an inexperienced observer (MLE(+)_inexp_) compared to the standard of reference of the results of an experienced observer (MLE(+)_exp_) demonstrated in 65% (28/43) false positive and in 5.6% (3/54) false negative results.

	MLE(+)_exp_	MLE(-)_exp_	Total
MLE(+)_inexp_	15	28	43
MLE(-)_inexp_	3	51	54
Total	18	79	97

## Results

**Figure 1:**
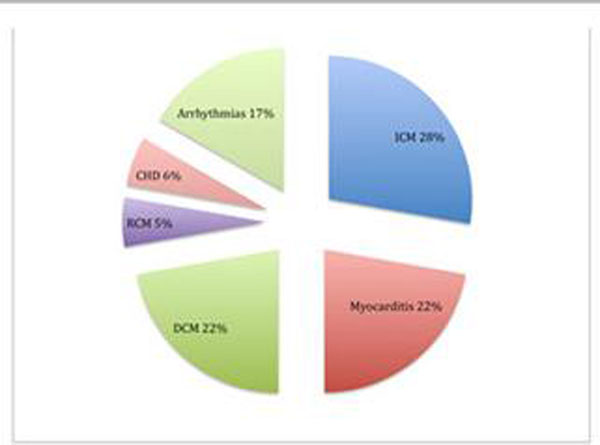
Pie chart shows the difference underlying cardiac diseases of patients with verified mid-wall late enhancement (MLE). As expected, the majority of patients with MLE presented with dilated cardiomyopathy (DCM) and myocarditis. But also in patients with ischemic cardiomyopathy (ICM), restrictive cardiomyopathy (RCM), congenital heart disease (CHD) and the occurrence of symptomatic arrhythmias without an underlying structural heart disease MLE could be detected.

**Figure 2:**
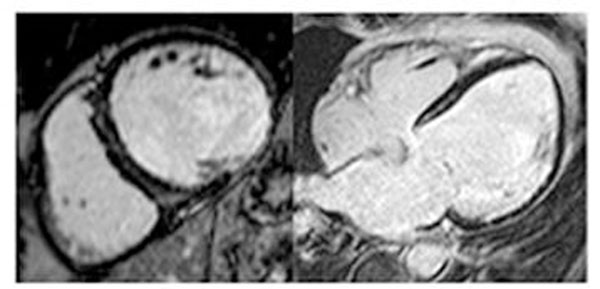
Patient with a DCM demonstrating with a typical MLE (arrow) is the basal land mid-ventricular anterseptal and interoseptal segments in the PSIR (SA) and IG-GRE (4CH) sequence.

**Figure 3:**
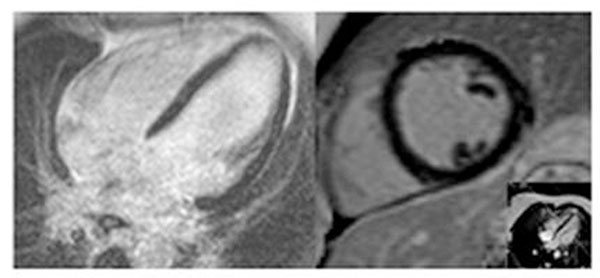
Patients with suspected myocarditis and false positive MLE as detected by an inexperienced observer due to a wrong inversion time (TI) in IR-GRE sequence (4CH) – arrows, which could be verified by a normal nulling of viable myocardium in the PSIR sequence (SA).

**Figure 4:**
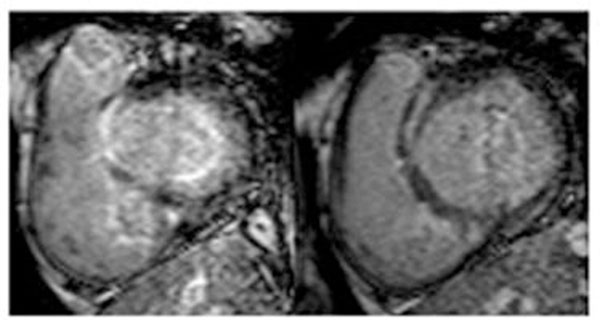
Partial volume effect (PVO) of the adjacent aorta and left ventricular outflow tract in the basal interoseptal segment mimmidding MLE (arrow).

The IO described suspected MLE in 43/97 patients (44%), which were false positive in 28/43 (65%). Only 18/97 (19%) were true MLE. Reasons for false positives were wrong TI in 39% (Table 2), PVE (25%), microvascular obstruction (MO) mimicking MLE in 11% and artifacts. The 3 false negative cases were interpreted in 2 cases as motion artifacts and overlooked in one case by the IO. As expected the majority of patients with MLE presented with DCM and myocarditis. But also in patients with ischemic cardiomyopathy (ICM), restrictive cardiomyopathy (RCM), congenital heart disease (CHD) and the occurrence of symptomatic arrhythmias without an underlying structural heart disease MLE could be detected. Figures 1, 2, 3, 4.

**Table 2:** Reasons for misinterpretation (False Positive Results) of increase myocardial signal intensity as MLE by the inexperienced observer were in the majority of cases a wrong inversion time (TI) following by partial volume effects (PVE), and trigger of breathing artifacts. Microvascular obstruction (MO) was also misinterpreted as MLE in three cases.

False Positive	
Reason	Number

Wrong TI	11/28
MO	3/28
PVE	7/28
Artifact	7/28

## Conclusion

MLE is a common finding not only in patients with DCM and myocarditis, but also in patients with ICM, RCM, CHD or patients with different arrhythmias without an underlying structural heart disease. Standardized criteria for the detection/definition of MLE are mandatory to reduce the number of false positive results, which can be higher than 50%, especially when cMRI is interpreted by an inexperienced cardiac MRI user.

